# Associations of educational attainment with Sepsis mediated by metabolism traits and smoking: a Mendelian randomization study

**DOI:** 10.3389/fpubh.2024.1330606

**Published:** 2024-02-01

**Authors:** Ying Lan, Lvlin Chen, Chao Huang, Xiaoyan Wang, Peng Pu

**Affiliations:** ^1^Department of Critical Care Medicine, Affiliated Hospital of Chengdu University, Chengdu, China; ^2^Department of Clinical Nutrition, The First Affiliated Hospital of Chengdu Medical College, Chengdu, China; ^3^Department of Cardiology, The First Affiliated Hospital of Chongqing Medical University, Chongqing, China

**Keywords:** education, Sepsis, mediation analyses, Mendelian randomization, body mass index

## Abstract

**Objective:**

Sepsis constitutes a significant global healthcare burden. Studies suggest a correlation between educational attainment and the likelihood of developing sepsis. Our goal was to utilize Mendelian randomization (MR) in order to examine the causal connection between educational achievement (EA) and sepsis, while measuring the mediating impacts of adjustable variables.

**Methods:**

We collected statistical data summarizing educational achievement (EA), mediators, and sepsis from genome-wide association studies (GWAS). Employing a two-sample Mendelian randomization (MR) approach, we calculated the causal impact of education on sepsis. Following this, we performed multivariable MR analyses to assess the mediation proportions of various mediators, including body mass index (BMI), smoking, omega-3 fatty acids, and apolipoprotein A-I(ApoA-I).

**Results:**

Genetic prediction of 1-SD (4.2 years) increase in educational attainment (EA) was negatively correlated with sepsis risk (OR = 0.83, 95% CI 0.71 to 0.96). Among the four identified mediators, ranked proportionally, they including BMI (38.8%), smoking (36.5%), ApoA-I (6.3%) and omega-3 (3.7%). These findings remained robust across a variety of sensitivity analyses.

**Conclusion:**

The findings of this study provided evidence for the potential preventive impact of EA on sepsis, which may be influenced by factors including and metabolic traits and smoking. Enhancing interventions targeting these factors may contribute to reducing the burden of sepsis.

## 1 Introduction

Sepsis is defined as a severe dysfunction of the body’s organs caused by the host response being disrupted due to an infection (1). Sepsis therefore burdens public health and the economy around the globe. According to a study conducted in 2017, approximately 48.9 million cases of sepsis were reported, resulting in 11.0 million fatalities related to sepsis, accounting for approximately 19.7% of all worldwide deaths (2, 3).

Education stands as the most potent indicator of socio-economic status, exerting influence over lifestyle choices and access to health resources (4). Although there have been several investigations highlighting the links between education and various illnesses (4–7), the relationship between educational achievement and sepsis has received comparatively less focus. Several studies have found that there is an inverse relationship between achieving higher levels of education and the mortality rates caused by sepsis (8, 9). Due to unknown or inadequately measured confounding factors, observational study results lack credibility in inferring causality. According to research, there are various factors that can partially influence the connection between education and different illnesses (10, 11). Sepsis has been associated with cardiovascular metabolic traits, obesity attributes, and lifestyle according to several studies (12–16). Nevertheless, the unexplored aspect is the degree to which these changeable factors elucidate the impact of education on sepsis. Understanding this topic can contribute to optimizing sepsis prevention at both clinical and public health levels.

The utilization of genetic variation as instrumental variables for causal inference between exposure and disease is a key aspect of Mendelian randomization (MR) analysis (17). The methodology employed is similar to that of a randomized controlled trial, which helps to reduce certain biases and reverse causality that are inherent in observational studies (18). For this research, we utilized a two-sample MR approach to examine the distinct causal connection between education and sepsis. Subsequently, we used multivariable MR to assess and quantify the mediating effects of these modifiable factors in sepsis.

## 2 Materials and methods

### 2.1 Study design

**Figure 1:**
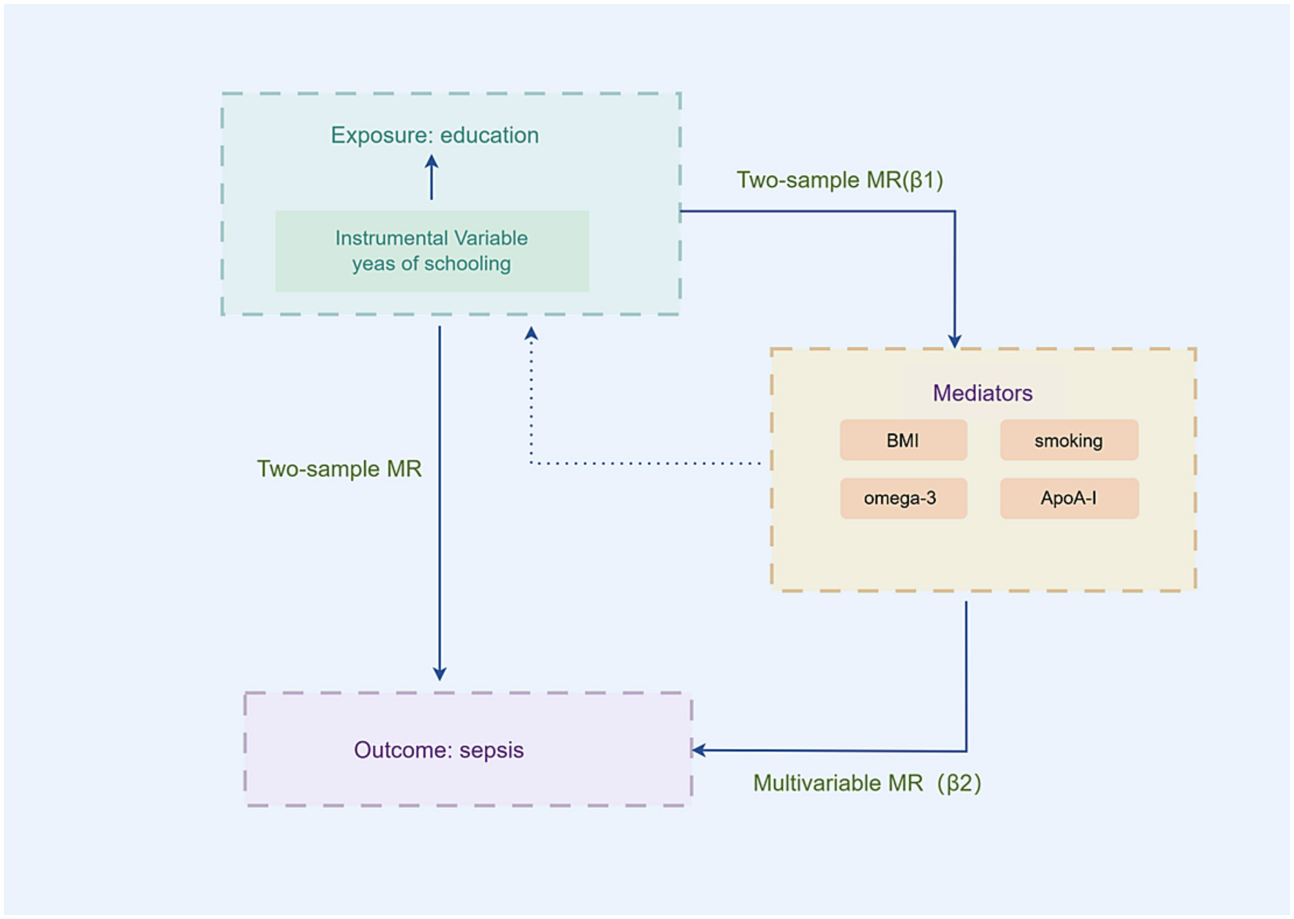
Overview of the study design.

This study comprised two phases of analysis (see Figure 1). Using the univariable Mendelian randomization (UVMR) analysis, we evaluated the connection between education and the risk of sepsis during the initial stage. During the second stage, potential mediators linked to education and sepsis were evaluated, and their mediating impacts were computed utilizing a two-step MR methodology. The study’s reporting followed the suggestions outlined in the Strengthening the Reporting of Observational Studies in Epidemiology (STROBE)-MR guidelines (18).

### 2.2 Data source

In this study, data for EA (educational attainment), mediators, and sepsis primarily stem from genome-wide association studies (GWAS) conducted in individuals of European ancestry. Table 1 contains all the condensed information.

**Table 1:** Summary of the GWAS data used in the MR analyses.

Phenotype	Unit	No of participants	Ancestry	Consortium/cohort	Year of publication	PubMed ID
*Exposure*						
Education (Years of schooling)	SD (4.2 y)	1,131,881	European	SSGAC	2018	30,038,396
*Outcome*						
Sepsis	Event	486,484	European	UK Biobank	2021	NA
*Mediators*						
BMI	SD	681,275	European	GIANT	2018	30,124,842
Cigarettes smoked per day	SD	249,752	European	GSCAN	2019	30,643,251
Omega-3	SD	114,999	European	NA	2020	NA
Apolipoprotein A1	SD	115,078	European	NA	2020	NA

BMI, body mass index; SD, standard deviation; SSGAC, Social Science Genetic Association Consortium; GIANT, Genetic Investigation of Anthropometric Traits; GSCAN, GWAS & Sequencing Consortium of Alcohol and Nicotine use.

#### 2.2.1 Exposures

Educational attainment was defined as the number of years of schooling completed by an individual. The genetic instruments for education were selected from a Genome-Wide Association Study (GWAS) on years of schooling conducted by the Social Science Genetic Association Consortium (SSGAC) on a sample of 1,131,881 individuals of European ancestry (19). Each standard deviation (SD) represents an increase of 4.2 years in educational attainment. The primary genetic instruments consisted of 371 independent SNPs (*r*^2^ < 0.001; distance threshold, 10,000 kb) that were genome-wide significant (*p* < 5 × 10^−8^).

#### 2.2.2 Mediators

We conducted two literature searches using the PubMed database. The first search identified previously published MR studies on sepsis and education. The second search identified studies investigating the association between education and sepsis (for specific search strategies, see Appendix S2 in Supplementary material). Finally, by combining these literature reviews, we identified 20 potential mediators that may underlie the impact of education on the risk of sepsis (for an overview of the process, please refer to Supplementary Figure S1). After considering the criteria for selecting a mediator, a total of 4 factors that can be changed (body mass index, omega-3 fatty acid, ApoA-I, and Cigarettes smoked per day) were included in the analysis of mediation. For detailed procedures, please refer to Supplementary Figure S1.

#### 2.2.3 Outcome

The GWAS conducted in the UK Biobank provided genetic associations for sepsis. The study involved 10,154 sepsis cases and 452,764 controls (Table 1).

This study used publicly available GWAS data. The data we used was summarized from published studies that had gained ethical approval and written consent from participants. The study did not require ethical approval.

### 2.3 Statistical analysis

#### 2.3.1 Effect of EA on Sepsis

To determine the overall influence of educational attainment (EA) on sepsis, we utilized a 2-sample Mendelian randomization (2-sample MR) technique. Our primary analytical methodology was the utilization of the Inverse Variance Weighted (IVW) approach. The findings were displayed as odds ratios (ORs) distributed across a 95% confidence interval (CI). Within the framework of the IVW approach, a significance level lower than *p* < 0.05 was deemed suggestive of possible connections.

#### 2.3.2 Mediation MR analysis

At first, we utilized a MR method that involved two samples to assess the impact (β1) of education on each mediator factor. Afterwards, by employing Multivariable Mendelian Randomization (MVMR), we calculated the causal impact (β2) of every mediator on the risk of sepsis, while taking into account education as a factor of adjustment. To evaluate the indirect impact, we employed the coefficient product approach, which entailed multiplying the outcomes of the two stages (β1 × β2). Afterwards, we calculated the ratio of the overall impact of education on sepsis that was influenced by each mediator individually, by dividing the indirect effect by the total effect. Standard errors were derived using the delta method, employing effect estimates obtained from the 2SMR analysis (20).

#### 2.3.3 MR sensitivity analysis

In the analysis of 2-sample MR, we utilized weighted median, MR Egger, and outlier techniques to evaluate the reliability of the IVW findings. Concurrently, we utilized MR Egger’s intercept to investigate pleiotropy and employed the Q′ heterogeneity statistic to assess heterogeneity. In addition, we performed reverse causation tests to investigate the impact of mediator variables on educational achievement.

The Two Sample MR and MVMR packages in R were used for all analyses.

## 3 Results

### 3.1 Effect of education on sepsis

The MR analysis provided strong evidence suggesting that education has a significant effect on the risk of sepsis (OR: 0.83, 95% CI: 0.71 to 0.96, *p* = 0.01) per SD increase in EA (Supplementary Table S3). Furthermore, there were no signs of heterogeneity or horizontal pleiotropy found (Supplementary Tables S4, S5).

### 3.2 Effect of EA on mediators

A total of 4 mediators were included in the MR analysis (Supplementary Figure S1). In the analysis of UVMR, there was a correlation between an increase of 1-SD in years of education and a decrease in BMI (β = −0.177 SD, 95% CI −0.226 to −0.128), −a reduction in smoking intensity (−0.334 SD, 95% CI −0.406 to −0.262), and higher levels of omega-3 (0.096SD, 95% CI 0.048 to 0.144) and ApoA-I (0.104SD, 95% CI 0.061 to 0.147) (Table 2). The genetic instrumental factors for education showed consistent heterogeneity and no pleiotropy with the mediator factors (See Supplementary Tables S6, S7). In the bidirectional MR analysis, a negative correlation was observed only between BMI and educational attainment, primarily driven by horizontal pleiotropy (Supplementary Table S8).

**Table 2:** UVMR assessing the causal association between education and each mediator.

Mediator	Method	No of SNPs	β (95% CI)	*p*-value
BMI	IVW	197	−0.177 (−0.226 to −0.128)	1.27e-12
	WM	197	−0.171 (−0.212 to −0.129)	1.20e-15
	MR Egger	197	−0.172 (−0.402 to 0.056)	1.41e-01
Cigarettes smoked per day	IVW	300	−0.334 (−0.406 to −0.262)	8.86e-20
	WM	300	−0.284 (−0.372 to −0.197)	1.88e-10
	MR Egger	300	−0.165 (−0.447 to 0.116)	2.50e-01
Omega-3	IVW	303	0.096 (0.048 to 0.144)	7.36e-05
	WM	303	0.086 (0.023 to 0.148)	6.91e-03
	MR Egger	303	0.101 (−0.087 to 0.288)	2.94e-01
ApoA-I	IVW	306	0.104 (0.061 to 0.147)	2.71e-06
	WM	306	0.082 (0.027 to 0.138)	3.41e-03
	MR Egger	306	0.079 (−0.092 to 0.251)	3.65e-01

BMI, body mass index; ApoA-I, Apolipoprotein; IVW, inverse variance weighted; WM, Weighted Median; OR, odds ratio; CI, confidence interval; the significance was at *p* < 0.05.

### 3.3 Effect of mediator on sepsis with adjustment for EA

After adjusting for education, each mediator was significantly associated with sepsis in the MVMR results. In particular, for every 1-SD increase in BMI (OR: 1.51, 95% CI: 1.37–1.65) and cigarettes smoked per day (OR: 1.23, 95% CI: 1.12–1.34). In contrast, each 1-SD increase in omega-3 levels (OR: 0.93, 95% CI: 0.87–0.99) and ApoA-I levels (OR: 0.89, 95% CI: 0.83–0.97) was associated with a reduced risk of sepsis after adjusting for education (Supplementary Table S9).

### 3.4 Individual proportion mediated

**Figure 2:**
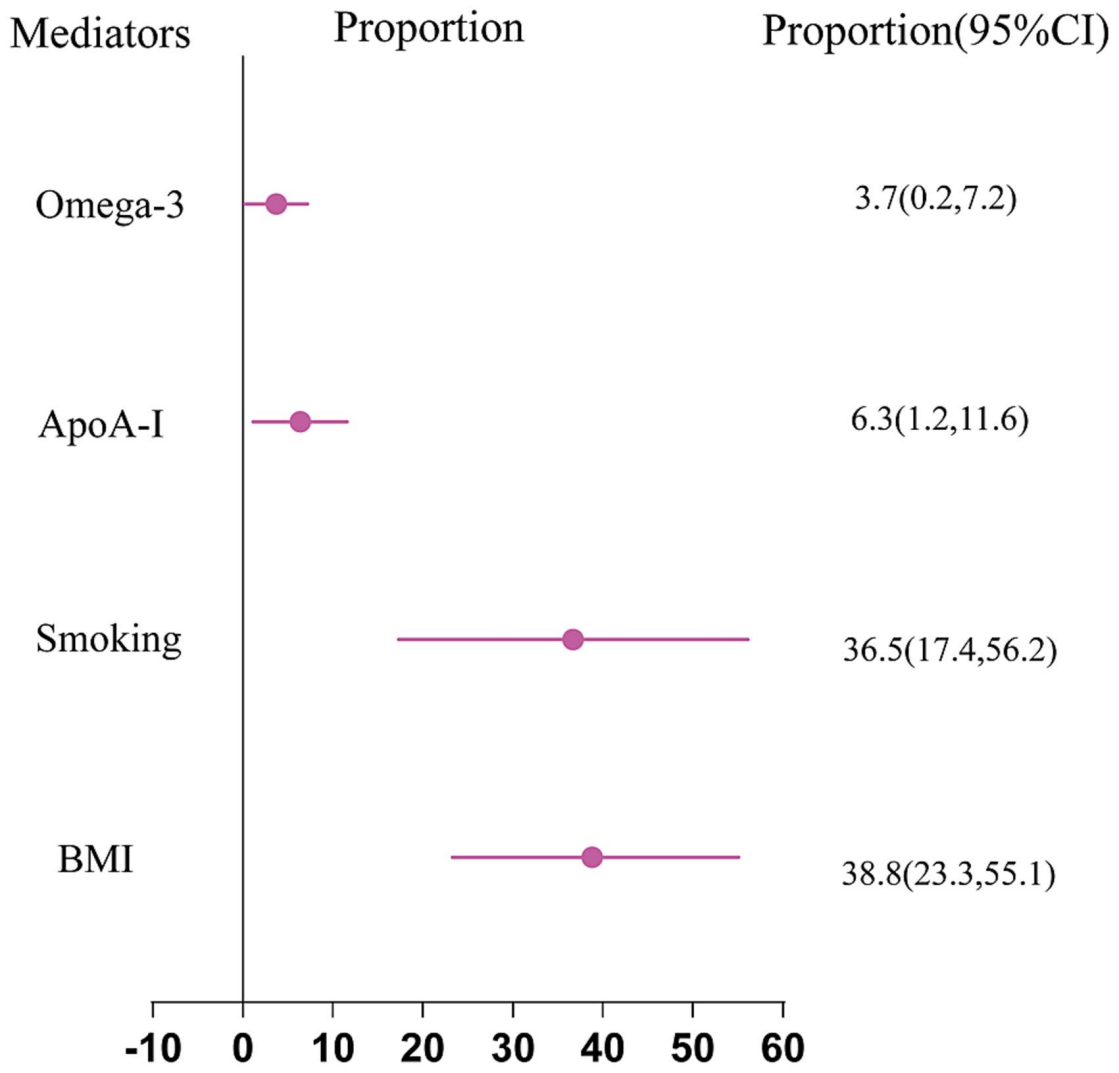
MR estimates of mediator-mediated proportions in the causal relationship between education and sepsis.

The mediator factors that were chosen, ranked by the proportion of mediation, are BMI (38.8%; [95% CI 23.3% ~ 55.1%]), smoking (36.5%; [95% CI 17.4–56.2%]), ApoA-I (6.3%; [95% CI 1.2–11.6%]) and omega-3 (3.7%; [95% CI 0.2–7.2%])according to Figure 2.

## 4 Discussion

Our MR study found evidence indicating both a causal and protective impact of education on sepsis. Each additional unit of education (equivalent to 4.2 years) resulted in a decrease of about 17% in the risk of sepsis. In addition, our research identified four factors as causal mediators in the pathway from education to sepsis, including BMI (38.8%), smoking (36.5%), ApoA-I (6.3%), and omega-3 (3.7%).Our study underscores the substantial mediating role of obesity-related traits and smoking in the pathogenesis from education to sepsis. Therefore, improving interventions aimed at these factors could potentially decrease the occurrence of sepsis.

Indeed, previous observational research has shown that increased levels of educational achievement act as a safeguard against sepsis. As reported by Wang et al. (21), lower educational levels are associated with a higher risk of sepsis (HR: 1.88; 95% CI, 1.54–2.29). However, the authors did not control for other confounding factors such as comorbidities, race, and health behaviors. Another cohort study using propensity scores (22), after accounting for other socioeconomic factors, individuals with a moderate level of education exhibited a higher risk of intensive care unit (ICU) admission due to sepsis when compared to those with a higher level of education. Furthermore, prior investigations suggested that within the ICU, sepsis patients with limited education displayed a tendency for early readmission (23). However, it is important to note that the majority of the information discussed here originated from observational studies, which may not have adequately considered residual confounding variables, thus lacking definitive establishment of causation. As far as we know, our MR analysis was the first to demonstrate that a 17% decrease in the risk of sepsis with each SD (equivalent to 4.2 years) increase in education attainment. Although education is usually achieved during early years, it has the potential to impact the desire for knowledge and well-being in later stages of life (24), thus leading to enhancements in lifestyle choices. Hence, potentially addressing issues of educational inequality and contributing to efforts in preventing sepsis and its associated health burdens.

Another notable finding in this research was the revelation of intermediary elements that clarify the connection between education and sepsis. During this investigation, a thorough examination revealed four factors that cause mediation. Intriguingly, obesity-related traits (BMI) and smoking, individually, mediated more than 35% of the overall mediation effects. Previously conducted observational studies (25, 26) and Mendelian research (12, 13, 27) have established a close association between obesity, smoking, and sepsis. Moreover, the HUNT investigation has revealed that the combined influence of smoking and alcohol intake contributes to 57% of the sepsis vulnerability linked to lower educational attainment (9). However, in our MR analysis, no causal relationship was discerned between alcohol consumption and sepsis. Furthermore, our MR study has discovered that omega-3 and ApoA-I, among the range of metabolic markers, have a causal role of 3.7 and 6.3% correspondingly, in influencing the risk of sepsis related to education. Remarkably, education maintains independent correlations with various metabolic parameters, including omega-3 fatty acids and Apolipoprotein A1, even after adjustment for BMI and smoking (28). This association could be partially explained by differences in eating patterns (28). Previous Mendelian research (27, 29) have shown that increased amounts of omega-3 and ApoA-I have the potential to reduce the risk of sepsis. The exact mechanisms are still unknown but could potentially include changes in the gut microbiome and increased production of substances that reduce inflammation (30–32). To summarize, the promotion of a healthier way of life, which includes consistent physical activity, healthy eating habits, and avoiding smoking, can result in substantial advantages for public health. Additionally, reinforcing public health education can help control the occurrence of sepsis.

To the best of our understanding, this study is the first Mendelian randomization attempt to uncover the causal influence of education on sepsis, while also identifying the causal factors that link education and sepsis. This endeavor possesses several notable strengths. By using SNPs as genetic instruments, biases caused by confounding and reverse causation are reduced, allowing for accurate causal estimates of the effect of education on outcomes. Its purpose is to remove the impact of elements like socioeconomic status and additional environmental factors. Additionally, we utilized the most recent data from the sepsis genome-wide association study (GWAS), which showed limited similarity with exposure or mediator. Furthermore, in our analysis comparing two samples, we conducted several sensitivity analyses to mitigate the effects of horizontal pleiotropy and biases originating from other factors, thereby showcasing the resilience of our research.

This study also carries certain limitations. Initially, because of limitations in database accessibility, certain vital variables, like age, sex, atmospheric contamination, and personal behavioral actions (for instance, sedentary behavior, watching television), were unable to be examined. Furthermore, the continuous existence of SNP heterogeneity could potentially introduce prejudice and affect the reliability of our MR findings. Additionally, the majority of the individuals involved in the study were of European origin. Therefore, it is important to be cautious when generalizing our research results to different ethnic populations or to countries with lower- and middle-income levels.

## 5 Conclusion

The findings of our research confirm that having a higher level of education reduces the risk of developing sepsis. This causal effect is partially mediated by obesity and smoking. It underscores the significant public health importance of enhancing the metabolism and lifestyle of individuals with lower education levels for the prevention of sepsis.

## Data availability statement

The original contributions presented in the study are included in the article/Supplementary material, further inquiries can be directed to the corresponding author.

## Author contributions

YL: Writing – original draft. LC: Writing – review & editing. CH: Writing – original draft. XW: Writing – original draft. PP: Writing – review & editing.
