# Supplementary material for: Associations of educational attainment with Sepsis mediated by metabolism traits and smoking: a Mendelian randomization study
Source: Front Public Health. 2024 Feb 1;12:1330606. doi: 10.3389/fpubh.2024.1330606 (PMC10867269; doi:10.3389/fpubh.2024.1330606)
Supplement: Supplementary file 1 [file Data_Sheet_1.docx]

Supplementary Material

**Appendix S1. List of** **candidate mediators identified from** **literature search.**

- Obesity-related characteristics (1, 2)

BMI(3)

Overweight

waist circumference(4)

- Lipid profiles (5, 6)

Low-density lipoprotein cholesterol (7)

High-density lipoprotein cholesterol

Total cholesterol

Apolipoprotein A1

- glucose metabolism-related traits

T2DM(8)

HbA1c(9)

Fasting glucose

- Physical activity or sedentary behavior(10)

Watching TV

Moderate to vigorous physical activity

- Stress-related trait

Major depression(11)

Insomnia(12)

- Lifestyle or dietary behavior(3)

Smoking heaviness (Cigarettes smoked per day)

Smoking initiation

Omega-3 fatty acid

Alcohol drinking

- Socioeconomic factor

Total household income

Air pollution exposure (PM2.5)(13)

**Reference**

1. Wang HE, Griffin R, Judd S, Shapiro NI, Safford MM. Obesity and risk of sepsis: a population-based cohort study. Obesity (Silver Spring). 2013;21(12):E762-9.

2. Ponsford MJ, Gkatzionis A, Walker VM, Grant AJ, Wootton RE, Moore LSP, et al. Cardiometabolic Traits, Sepsis, and Severe COVID-19: A Mendelian Randomization Investigation. Circulation. 2020;142(18):1791-3.

3. Paulsen J, Askim A, Mohus RM, Mehl A, Dewan A, Solligard E, et al. Associations of obesity and lifestyle with the risk and mortality of bloodstream infection in a general population: a 15-year follow-up of 64 027 individuals in the HUNT Study. Int J Epidemiol. 2017;46(5):1573-81.

4. Gurunathan U, Rapchuk IL, Dickfos M, Larsen P, Forbes A, Martin C, et al. Association of Obesity With Septic Complications After Major Abdominal Surgery: A Secondary Analysis of the RELIEF Randomized Clinical Trial. JAMA Netw Open. 2019;2(11):e1916345.

5. Hofmaenner DA, Arina P, Kleyman A, Page Black L, Salomao R, Tanaka S, et al. Association Between Hypocholesterolemia and Mortality in Critically Ill Patients With Sepsis: A Systematic Review and Meta-Analysis. Crit Care Explor. 2023;5(2):e0860.

6. Lou C, Meng Z, Shi YY, Zheng R, Qian SZ, Pan J. Genetic association of lipids and lipid-lowering drugs with sepsis: a Mendelian randomization and mediation analysis. Front Cardiovasc Med. 2023;10:1217922.

7. Guirgis FW, Donnelly JP, Dodani S, Howard G, Safford MM, Levitan EB, et al. Cholesterol levels and long-term rates of community-acquired sepsis. Crit Care. 2016;20(1):408.

8. Zohar Y, Zilberman Itskovich S, Koren S, Zaidenstein R, Marchaim D, Koren R. The association of diabetes and hyperglycemia with sepsis outcomes: a population-based cohort analysis. Intern Emerg Med. 2021;16(3):719-28.

9. Balintescu A, Lind M, Franko MA, Oldner A, Cronhjort M, Svensson AM, et al. Glycemic Control and Risk of Sepsis and Subsequent Mortality in Type 2 Diabetes. Diabetes Care. 2022;45(1):127-33.

10. Wang HE, Baddley J, Griffin RL, Judd S, Howard G, Donnelly JP, et al. Physical inactivity and long-term rates of community-acquired sepsis. Prev Med. 2014;65:58-64.

11. Askim A, Gustad LT, Paulsen J, Reitan SK, Mehl A, Mohus RM, et al. Anxiety and Depression Symptoms in a General Population and Future Risk of Bloodstream Infection: The HUNT Study. Psychosom Med. 2018;80(7):673-9.

12. Thorkildsen MS, Laugsand LE, Nilsen TIL, Mohus RM, Hovik LH, Rogne T, et al. Insomnia symptoms and risk of bloodstream infections: prospective data from the prospective population-based Nord-Trondelag Health Study (HUNT), Norway. J Sleep Res. 2023;32(1):e13696.

13. Honda TJ, Kazemiparkouhi F, Henry TD, Suh HH. Long-term PM(2.5) exposure and sepsis mortality in a US medicare cohort. BMC Public Health. 2022;22(1):1214.

**Appendix S2 PubMed search terms for literature review.**

- For identifying previous MR studies of sepsis, we used the following search terms:

((((((sepsis [Title/Abstract]) OR (septic shock [Title/Abstract])) OR (Septicemia [Title/Abstract])) OR (Endotoxemia [Title/Abstract])) OR (infectious disease [Title/Abstract])) OR (bloodstream infection [Title/Abstract])) AND ((mendelian randomization [Title/Abstract]) OR (mendelian randomization analysis [Title/Abstract]))

- For identifying previous MR studies of EA, we used the following search terms:

 ((((education attainment [Title/Abstract]) OR (education [Title/Abstract])) OR (Socioeconomic status [Title/Abstract])) OR (Socioeconomic [Title/Abstract])) AND ((mendelian randomization [Title/Abstract]) OR (mendelian randomization analysis [Title/Abstract]))

- For identifying the studies related to the relationship between EA and sepsis, we used the following search terms:

((((education attainment [Title/Abstract]) OR (education [Title/Abstract])) OR (Socioeconomic status [Title/Abstract])) OR (Socioeconomic [Title/Abstract])) AND (((((Septicemia [Title/Abstract]) OR (Endotoxemia [Title/Abstract])) OR (sepsis [Title/Abstract])) OR (septic shock [Title/Abstract])) OR (severe sepsis [Title/Abstract]))

**Table S2.** Summary of the GWAS Data with excluded mediator**.**

| **Phenotype** | **No of**  **participants** | **Ancestry** | **Consortium/**  **cohort** | **Year of**  **publication** | **PubMed ID** |
| --- | --- | --- | --- | --- | --- |
| Waist circumference | 231 353 | European | GIANT | 2015 | 25673412 |
| LDL-C | 173 082 | Mixed | GLGC | 2013 | 24097068 |
| HDL-C | 187 167 | Mixed | GLGC | 2013 | 24097068 |
| Total cholesterol | 187 365 | Mixed | GLGC | 2013 | 24097068 |
| T2DM | 149,821 | Mixed | DIAGRAMplusMetabochip | 2012 | 22885922 |
| HbA1c | 46,368 | European | MAGIC | 2010 | 20858683 |
| Fasting glucose | 58 074 | European | MAGIC | 2012 | 22885924 |
| Watching TV | 408 815 | European | UK Biobank | 2020 | 32317632 |
| MVPA | 377 234 | European | UK Biobank | 2018 | 29899525 |
| Major depression | 500 199 | European | PGC | 2019 | 30718901 |
| insomina | 371 145 | European | Finngen | NA | NA |
| Smoking initiation | 607 291 | European | GSCAN | 2019 | 30643251 |
| Alcohol drinking | 335,394 | European | GSCAN | 2019 | 30643251 |
| PM2.5 | 423,796 | European | UK Biobank | 2018 | 30643251 |
| Total household income | 397 751 | European | UK Biobank | 2018 | 29846171 |

LDL-C, Low-density lipoprotein cholesterol; HDL-C, High-density lipoprotein cholesterol; MVPA, Moderate to vigorous physical activity; PM2.5, particulate matter 2.5

**
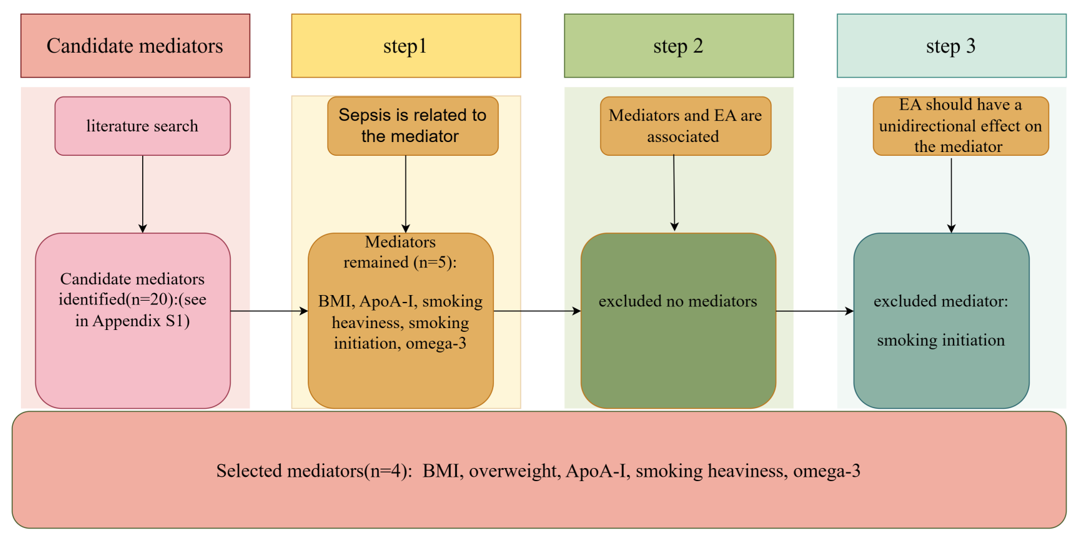
**

**Figure S1** an overview of screening the mediators. In step 1, we assessed the causal associations of mediators with sepsis using univariable Mendelian randomization (UVMR). In step 2, we first screened candidate mediators for the association between education and sepsis using 2-sample MR. In step 3, we excluded the mediator that has a bidirectional effect on education using 2S MR. BMI, body mass index; ApoA-I, Apolipoprotein A1.

**Table S3.** UVMR estimating the associations of education and sepsis.

| **Exposure** | **Outcome** | **Method** | **No. of SNPs** | **OR（95%CI）** | **P value** |
| --- | --- | --- | --- | --- | --- |
| Education | Sepsis | MR Egger | 246 | 0.89 (0.47 -1.67) | 0.71 |
|  |  | Weighted median | 246 | 0.85 (0.68 -1.06) | 0.13 |
|  |  | Inverse variance weighted | 246 | 0.83 (0.71 -0.96) | 0.01 |
|  |  | Simple mode | 246 | 0.83 (0.40-1.71) | 0.62 |
|  |  | Weighted mode | 246 | 0.83 (0.43-1.61) | 0.58 |

**Table S4.** MR heterogeneity test of the associations of education and Sepsis.

| **Exposure** | **Outcome** | **Method** | **Q statistic** | **Q df** | **Q p-value** |
| --- | --- | --- | --- | --- | --- |
| Education | Sepsis | IVW | 245.57 | 245 | 0.47 |
|  |  | MR Egger | 245.52 | 244 | 0.46 |

IVW, Inverse Variance Weighted; df, degree of freedom;

**Table S5.** MR directional pleiotropy test (MR Egger) of the associations of education and sepsis.

| **Exposure** | **Outcome** | **Egger intercept** | **SE** | **P value** |
| --- | --- | --- | --- | --- |
| Education | Sepsis | -0.00089 | 0.0042 | 0.83 |

**Table S6.** MR heterogeneity test of the association of education with each mediator

| **Exposure** | **Mediator** | **Method** | **Q statistic** | **Q df** | **Q p-value** |
| --- | --- | --- | --- | --- | --- |
| Education | BMI | IVW | 1058.69 | 196 | 2.59e-118 |
|  |  | MR Egger | 1058.68 | 195 | 1.11e-118 |
|  | Omega-3 | IVW | 465.89 | 302 | 4.01e-09 |
|  |  | MR Egger | 465.89 | 301 | 3.20e-09 |
|  | Cigarettes smoked per day | IVW | 568.25 | 299 | 5.97e-19 |
|  |  | MR Egger | 565.42 | 298 | 8.49e-19 |
|  | ApoA-I | IVW | 457.92 | 305 | 3.17e-08 |
|  |  | MR Egger | 457.79 | 304 | 2.62e-08 |

IVW, Inverse Variance Weighted; ApoA-I, Apolipoprotein A1.

**Table S7.** MR directional pleiotropy test (MR Egger) of the association of education with each mediator.

| **Exposure** | **Mediator** | **Egger intercept** | **SE** | **P-value** |
| --- | --- | --- | --- | --- |
| Education | BMI | -6.48e-05 | 0.0015 | 0.96 |
|  | Omega-3 | -5.87e-05 | 0.0013 | 0.96 |
|  | Cigarettes smoked per day | -0.0023 | 0.0019 | 0.22 |
|  | ApoA-I | 0.00034 | 0.0011 | 0.77 |

BMI, body mass index; ApoA-I, Apolipoprotein A1.

**Table S8.** Reverse MR estimating the association of each mediator with education

| **Mediator** | **Method** | **MR results** | | | **Heterogeneity test** | | **Directional pleiotropy test** | |
| --- | --- | --- | --- | --- | --- | --- | --- | --- |
|  |  | **β** | **SE** | **P value** | **Q statistic** | **Qp-value** | **Egger intercept** | **P value** |
| BMI | IVW | -0.107 | 0.011 | 8.67E-22 | 1913.96 | 7E-175 | -0.0018 | 0.00011 |
|  | Weighted Median | -0.086 | 0.011 | 1.37E-13 | NA | NA |  |  |
|  | MR Egger | -0.002 | 0.029 | 0.93 | 1853.22 | 3.2E-165 |  |  |
| Omega-3 | IVW | 0.007 | 0.004 | 0.11 | 67.83 | 0.009 | -0.0002 | 0.66 |
|  | Weighted Median | 0.003 | 0.005 | 0.44 | NA | NA |  |  |
|  | MR Egger | 0.011 | 0.006 | 0.15 | 67.52 | 0.007 |  |  |
| Cigarettes smoked per day | IVW | -0.018 | 0.012 | 0.15 | 59.25 | 1.39E-06 | -0.0048 | 0.0002 |
|  | Weighted Median | -0.001 | 0.008 | 0.93 | NA | NA |  |  |
|  | MR Egger | 0.037 | 0.014 | 0.02 | 25.41 | 0.062 |  |  |
| ApoA-I | IVW | 0.007 | 0.007 | 0.26 | 145.21 | 1.57e-07 | 0.0015 | 0.0162 |
|  | Weighted Median | -0.001 | 0.007 | 0.85 | NA | NA |  |  |
|  | MR Egger | -0.014 | 0.011 | 0.21 | 133.13 | 2.81e-06 |  |  |

BMI, body mass index; ApoA-I, Apolipoprotein A1; IVW, Inverse Variance Weighted.

**TABLE S9** MVMR Assessing the Causal Association Between Each Mediator and Sepsis with Adjustment for Education

| **Mediator** | **β(95%CI)** | **SE** | **OR (95% CI)** | **P value** |
| --- | --- | --- | --- | --- |
| BMI | 0.41（0.32-0.51） | 0.046 | 1.51(1.37-1.65) | 4.66E-19 |
| Omega-3 | 0.21（0.11-0.29） | 0.046 | 1.23(1.12-1.34) | 6.71E-06 |
| Cigarettes smoked per day | -0.07(-0.13 to -0.01) | 0.033 | 0.93(0.87- 0.99) | 0.03 |
| Apolipoprotein A1 | -0.11（-0.18 to -0.02） | 0.041 | 0.89（0.83-0.97） | 0.008 |
